# Head-to-head comparison of ^68^Ga-FAPI-04 PET/CT and ^18^F-FDG PET/CT in the evaluation of primary digestive system cancer: a systematic review and meta-analysis

**DOI:** 10.3389/fonc.2023.1202505

**Published:** 2023-06-26

**Authors:** Jiqi Ouyang, Peiwen Ding, Runshun Zhang, Yuexia Lu

**Affiliations:** ^1^ Department of Gastroenterology, Guang’anmen Hospital, China Academy of Chinese Medical Sciences, Beijing, China; ^2^ Graduate School of China Academy of Traditional Chinese Medicine, Beijing, China; ^3^ Clinical School, Chengdu University of Traditional Chinese Medicine, Chengdu, Sichuan, China; ^4^ Department of Oncology, Hospital of Chengdu University of Traditional Chinese Medicine, Chengdu, Sichuan, China

**Keywords:** 68 Ga-FAPI-04, 18 F-FDG, PET/CT, primary cancer, digestive system, meta-analysis

## Abstract

**Introduction:**

Althoug ^18^F-FDG positron emission tomography/computed tomography (PET/CT) is widely accepted as a diagnostic tool for detecting digestive cancers, ^68^Ga-FAPI-04 PET/CT may perform better in detecting gastrointestinal malignancies at an earlier stage. This study aimed to systematically review the diagnostic performance of ^68^Ga-FAPI-04 PET/CT compared with that of ^18^F-FDG PET/CT in primary digestive system cancers.

**Methods:**

In this study, a comprehensive search using the PubMed, EMBASE, and Web of Science databases was performed to identify studies that met the eligibility criteria from the beginning of the databases to March 2023. The quality of the relevant studies with the Quality Assessment of Diagnostic Accuracy Studies (QUADAS-2) method was assessed using the RevMan 5.3 software. Sensitivity and specificity were calculated using bivariate random-effects models, and heterogeneity was assessed with the I^2^ statistic and meta-regression analysis using the R 4.22 software.

**Results:**

A total of 800 publications were identified in the initial search. Finally, 15 studies comprising 383 patients were included in the analysis. The pooled sensitivity and specificity of ^68^Ga-FAPI-04 PET/CT were 0.98 (95% CI, 0.94–1.00) and 0.81 (95% CI, 0.23–1.00), whereas those of ^18^F-FDG PET/CT were 0.73 (95% CI, 0.60–0.84) and 0.77 (95% CI, 0.52–0.95), respectively. ^68^Ga-FAPI-04 PET/CT performed better for specific tumours, particularly in gastric, liver, biliary tract, and pancreatic cancers. Both imaging modalities had essentially the same diagnostic efficacy in colorectal cancer.

**Conclusions:**

^68^Ga-FAPI-04 PET/CT showed a higher diagnostic ability than ^18^F-FDG PET/CT in terms of diagnosing primary digestive tract cancers, especially gastric, liver, biliary tract, and pancreatic cancers. The certainty of the evidence was high due to the moderately low risk of bias and low concern regarding applicability. However, the sample size of the included studies was small and heterogeneous. More high-quality prospective studies are needed to obtain higher-quality evidence in the future.

**Systematic Review Registration:**

The systematic review was registered in PROSPERO [CRD42023402892].

## 1 Introduction

Digestive system cancer affects the largest number of organs and is widely distributed (1). According to the GLOBOCAN 2020 report (2), cancers of the digestive system are a significant global health burden, with colon cancer ranking third (10.1%) and gastric cancer (GC) ranking fifth (5.6%) among the most prevalent cancers. In China (3), four of the top five cancers associated with death are digestive tract tumours, namely, cancers of the liver (12.85%), stomach (12.48%), oesophagus (10.09%), and colorectum (9.63%). Despite this, early detection of digestive tract cancers remains an unmet clinical need (4). Therefore, it is critical to investigate personalized ways of identifying primary digestive tract cancers early, thereby establishing the best treatment approach for minimizing mortality (5).

The current imaging-based diagnostic modalities for tumours combined with pathology as the gold standard include ultrasound for thyroid cancer (6), mammography for breast cancer (7), and intraoperative ultrasound for colorectal cancer (8), whereas magnetic resonance imaging (MRI) is becoming the gold standard for liver (9) and prostate cancer (10) metastases. Traditional imaging methods, including endoscopic, ultrasound, computed tomography (CT), and MRI (11), are commonly recommended for the detection of primary digestive tract malignancies. However, these methods have certain limitations. For example, enhanced CT or MRI can fail to accurately distinguish small nodules from atypical lesions in patients with hepatocellular carcinoma (HCC) (12). Similarly, GC may not be detected during endoscopy (13), and colonoscopy may not always reach the caecum (14). Therefore, there is a need for a diagnostic tool that can identify every malignant tumour while minimizing false-positive findings (15).

Although histopathology remains the diagnostic gold standard, recent developments in imaging methods for evaluating cancers have made the non-invasive diagnosis of cancer possible (16). Positron emission tomography (PET) has played a significant role in the field of molecular imaging over the past decade (17) and is commonly utilized for cancer detection (18). The combined use of PET and CT can avoid the limitations of using each modality alone (19). A major advantage of PET/CT is that it can detect active lesions throughout the body and has a higher physical sensitivity than other commonly used imaging techniques (20). In the past 30 years, ^18^F-FDG tracers, which take advantage of the tumours’ aberrant glucose metabolism, have become increasingly available and are now the most widely used PET imaging tool. ^18^F-FDG-PET is frequently used to diagnose malignancies, evaluate the effectiveness of tumour treatment, and predict prognosis (21). However, recent research (22) has revealed that FDG tracers have limitations in the diagnosis of various gastrointestinal cancers and are unable to differentiate between inflammation and malignancy. Recent studies have also uncovered a correlation between increased fibroblast activation protein (FAP) levels in cancer-associated fibroblasts and tumour growth, metastasis, and prognosis. Fibroblast activation protein inhibitor (FAPI) has therefore emerged as a new cancer imaging molecule (23). Researchers are seeking radionuclides like ^68^Ga, ^18^F, ^99m^Tc, and ^111^In and FAPI derivatives with a better affinity for FAPI (24). Numerous findings for FAPI-04 in preclinical and clinical settings indicate the potential of FAP tracers for future theranostic applications, as their tumour uptake is quicker than that previously discovered for FAPI-02. However, fewer clinical trials have used the recently discovered FAPI-46, 34, 74, DOTA-2P(FAPI)2, and DOTA.SA (25). In this systematic review, we found plenty of research on the use of ^68^Ga-FAPI-04 PET/CT in gastrointestinal cancers. For instance, Pang et al. (26) reported that ^68^Ga-FAPI-04 PET/CT had higher sensitivity but lower specificity for primary digestive tract cancers than ^18^F-FDG PET/CT, whereas Lin et al. (27) found no difference in sensitivity between the two tracers.

Given these conflicting observations, there is currently a debate about whether ^68^Ga-FAPI-04 PET/CT is more sensitive than ^18^F-FDG PET/CT for the diagnosis of primary digestive tract tumours. To draw a more definitive conclusion, this systematic review with meta-analysis was conducted by collecting and analysing all the published studies that met the relevant eligibility criteria.

## 2 Methods

### 2.1 Search strategy

Three English electronic databases (PubMed, EMBASE, and Web of Science) were comprehensively and systematically searched from their inception to March 2023 using the following terms: 1) PET OR positron emission tomography, 2) ^68^Ga-FAPI OR FAPI-04 OR FAPI OR fibroblast activation protein OR FAP, and 3) Digestive OR Gastric OR Gastrointestinal OR Pancreatic OR Pancreas OR Pancreatic OR Colorectal OR Hepatic OR Hepatocellular OR Liver. The detailed search terms used are reported in **Table S1** in the Supplementary Material.

### 2.2 Eligibility criteria

Selection criteria were developed based on the principles of PICOS (Participants, Interventions, Comparisons, Outcomes, and Study design). Studies that matched all of the following criteria were considered: 1) participants: patients with digestive system tumours; 2) intervention: a head-to-head comparison of ^68^Ga-FAPI-04 PET/CT; 3) comparisons: ^18^F-FDG PET/CT; 4) gold standard: histological pathology or follow-up imaging (5); type of study: prospective or retrospective diagnostic studies; 6) language: studies published in English.

We excluded studies that were 1) duplicated papers; 2) abstracts, editorial comments, letters, case reports, reviews, or meta-analyses; 3) irrelevant studies; 4) studies in languages other than English; 5) studies in which the true-positive (TP), false-positive (FP), true-negative (TN), and false-negative (FN) data could not be extracted.

### 2.3 Data screening and extraction process

Two authors (J.O. and P.D.) performed the initial screening by reviewing the titles and abstracts of the records in Endnote X20. They were then assigned to conduct a secondary screening by independently reading the identified full text based on predetermined inclusion criteria. They also independently extracted data from the included studies using a Microsoft Excel spreadsheet. The extracted data included 1) author names and year of publication; 2) study characteristics, including country, design, analysis, and criteria for final diagnosis; 3) patient characteristics, including sample size, mean/median age, gender (M:F), and tumour type, size, and stage; 4) technical characteristics, including mean injected activity per kg or total for FAPI or FDG, time interval FAPI or FDG tracer injection and image acquisition, the median period between FAPI and FDG tracer, scanner modality, and TP, FP, FN, and TN. Disagreements that emerged during the screening process were left to the third author (S.Z.) to make the final decision based on the conditions included in the meta-analysis.

### 2.4 Risk of bias and quality assessment

Two qualified researchers (J.O. and Y.L.) used the Quality Assessment of Diagnostic Accuracy Studies (QUADAS-2) technique to analyze each study’s bias risk and applicability. The four main sections of this instrument cover “Patient Selection, Index Test, Reference Standard, and Flow and Timing”. Each domain is evaluated separately for risk of bias and includes three components: information used to support the judgment of risk of bias, signaling questions, and judgment of risk of bias. The questions are judged by “yes”, “no”, or “unclear”, where “yes” represents a low risk of bias. The domains other than “Timing” results were used to evaluate the applicability concerns, and each question was given a “low”, “high”, or “unclear” rating (28). The RevMan (version 5.3) software was used for the evaluation. An additional reviewer was engaged to resolve any potential disagreements.

### 2.5 Publication bias

To assess publication bias, an Egger’s test and a funnel plot were used. Statistical analyses were run with the R 4.2.2 statistical computing and graphics package. P-Values <0.05 were considered statistically significant.

### 2.6 Data synthesis

Using the DerSimonian and Laird method, the Freeman–Tukey double inverse sine transformation was used to evaluate and transform sensitivities and specificities. Jackson’s method was used to calculate confidence intervals. Our analysis of heterogeneity within and between groups was based on the Cochrane Q and I^2^ statistics. We decided to perform a sensitivity analysis if there was a substantial difference in study heterogeneity (p < 0.10 or I^2^ > 50%) by reassessing the sensitivities and specificities after excluding each publication individually. Furthermore, a meta-regression analysis was performed if the sensitivity analysis could not identify any sources of heterogeneity. These analyses were conducted to determine the robustness of the overall sensitivities and specificities and to identify single studies that may contribute to heterogeneity.

## 3 Results

### 3.1 Literature search

**Figure 1:**
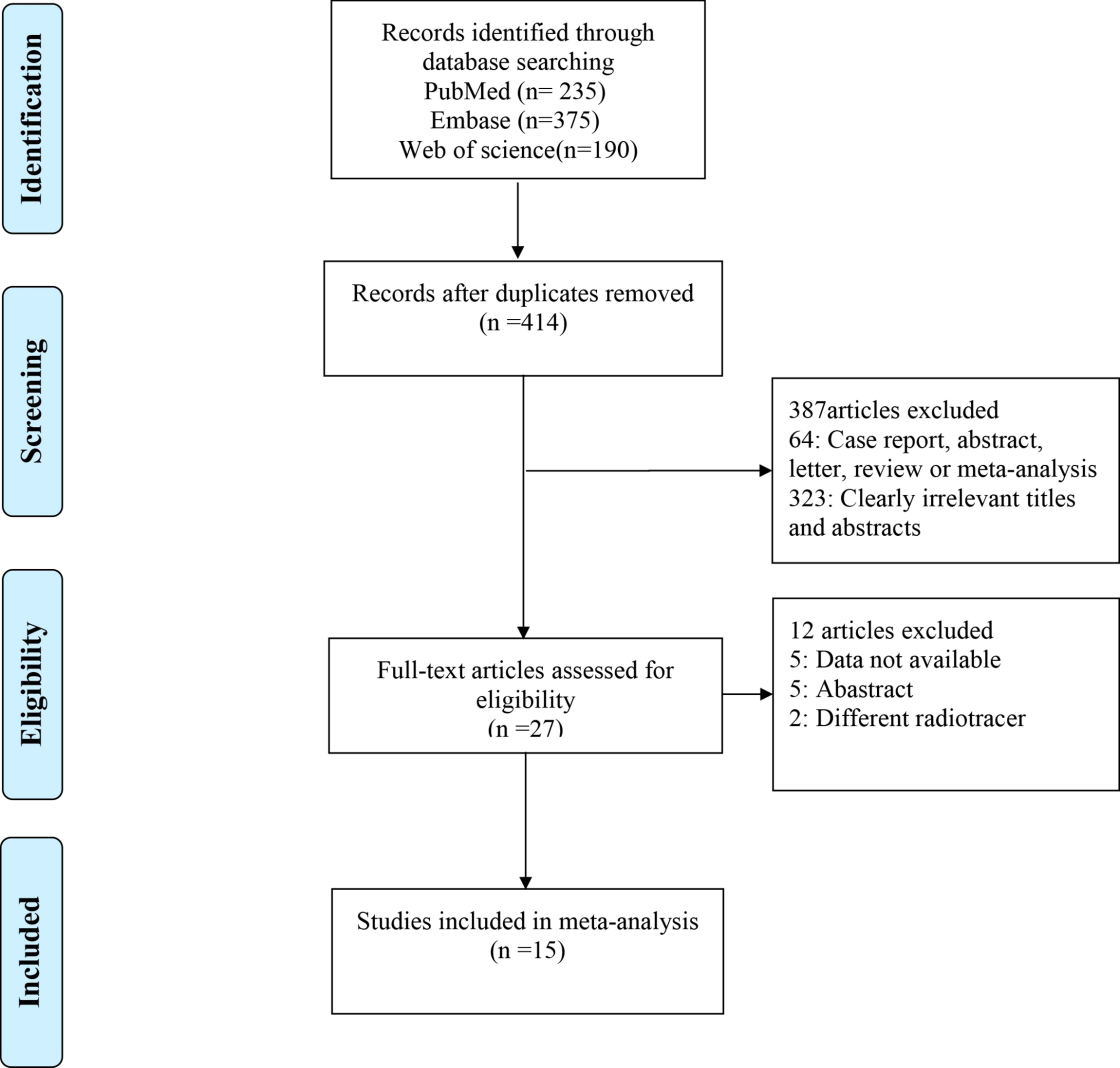
The PRISMA flow chart of the study selection process. PRISMA, Preferred Reporting Items for Systematic Reviews and Meta-Analyses.

The initial search yielded 800 publications, of which 414 remained after eliminating 386 duplicates. Of these, 387 studies were excluded based on their title or abstract, with 64 being case reports, abstracts, letters, reviews, or meta-analyses, and 323 having irrelevant titles and abstracts. Of the remaining 27 studies, five lacked data, five were abstracts, and two used different radiotracers; these were therefore excluded. Finally, 15 studies were included, which evaluated head-to-head the diagnostic performance of ^68^Ga-FAPI-04 PET/CT and ^18^F-FDG PET/CT for primary digestive system cancer. **Figure 1** shows the Preferred Reporting Items for Systematic Reviews and Meta-Analyses (PRISMA) flow diagram for selecting the included studies.

### 3.2 Characteristics of the included studies

**Table 1** summarizes the characteristics of the 15 studies that were included in this meta-analysis. The included studies involved a total of 383 participants with primary gastrointestinal cancers. The studies were published between 2020 and 2023 and included patients with five different main types of cancer: six for GC (26, 29–34), four for liver cancer (35–38), three for biliary tract carcinoma (BTC) (37–39), four for colorectal cancer (26, 27, 34, 40), and one for pancreatic cancer (41). Of these 15 studies, eight were retrospective and seven were prospective. Four studies used pathological diagnosis alone as the gold standard, and 11 used either pathological diagnosis or imaging follow-up. Ten studies were based on patient analysis, while five were based on lesion analysis. We derived the following: the mean age of the included patients from four studies, with one study reporting a mean age of <60 years and four studies reporting a mean age of ≥60 years; the tumour size from five studies, with two studies reporting a tumour size of <3 cm and three with a tumour size ≥3 cm; the patients’ gender distribution from six studies, with men accounting for <70% in three studies and ≥70% in the other three studies; and the tumour stage from seven papers, with five studies reporting early and advanced stages with a ratio of <1 and two studies with a ratio of ≥1. Other technical aspects are displayed in **Table S2** in the Supplementary Material.

**Table 1:** Study and PB characteristics of the included studies.

Authors, years	Country	Study design	Age (range)	Gender (M%)	No. of patients	Criteria	Analysis	Type	Size (cm)	Stage (early: advanced)
(26) 2021	China	Retro	NA	NA	19	PA	PB	GC, CRC	2.2	21:7
(27) 2023	China	Pro	NA	NA	36	PA or FU	LB	CRC	NA	NA
(29) 2022	China	Retro	NA	NA	22	PA or FU	PB	GC	1.6	13:9
(30) 2021	Turkey	Pro	60.5	60%	15	PA	PB	GC	NA	NA
(31) 2022	China	Pro	64	71%	62	PA or FU	LB	GC	>3 cm 52 ≤3 cm 10	8:54
(32) 2022	China	Pro	NA	NA	45	PA or FU	PB	GC	NA	35%:68.75%
(33) 2022	China	Retro	NA	52.6%	19	PA or FU	PB	GC	NA	8:11
(34) 2022	China	Retro	NA	NA	18	PA or FU	PB	GC, CRC	NA	NA
(35) 2021	China	Retro	NA	96%	25	PA	LB	HCC	NA	21:7
(36) 2020	China	Pro	NA	NA	17	PA or FU	PB	HCC	NA	NA
(37) 2020	China	Retro	NA	NA	23	PA or FU	PB	HCC, BTC	4.57	NA
(38) 2023	Thailand	Retro	68	71%	27	PA or FU	PB	HCC, BTC	NA	NA
(39) 2022	China	Pro	61.8	NA	13	PA or FU	LB	BTC	NA	2:14
(40) 2023	India	Retro	NA	NA	16	PA	LB	CRC	NA	NA
(41) 2021	China	Pro	NA	61.5%	26	PA or FU	PB	PCAN	3.9	NA

Type, tumour type; Size, tumour size; Stage, tumour stage; Pro, prospective; Retro, retrospective; PB, patient-based; LB, lesion-based; PA, pathology; FU, follow-up; GC, gastric cancer; CRC, colorectal cancer; HCC, hepatic cell carcinoma; BTC, biliary tract carcinoma; PCAN, pancreatic cancer; NA, not available.

### 3.3 Risk of bias and quality assessment

**Figure 2:**
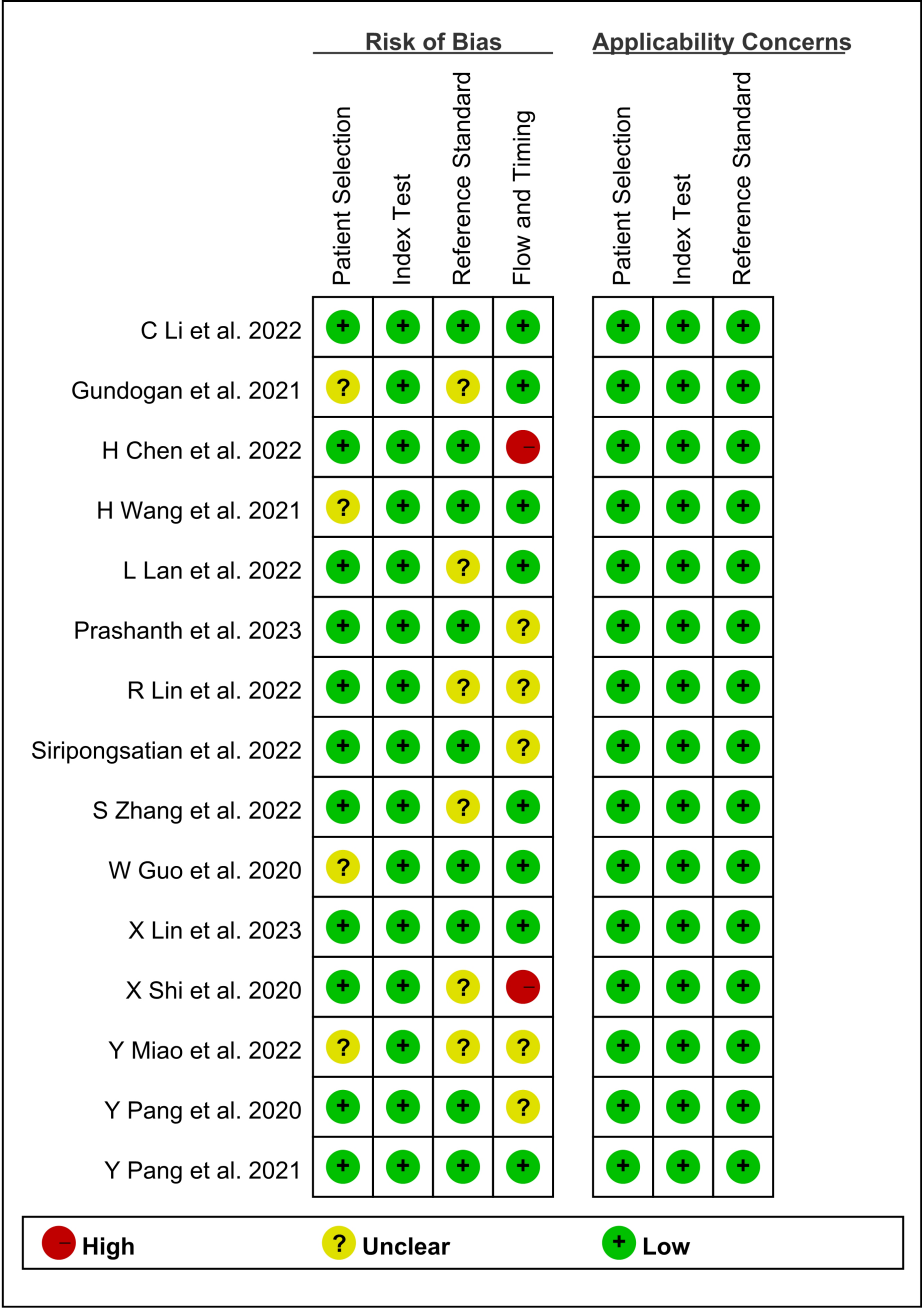
Graph of risk of bias and applicability of all eligible studies based on the QUADAS-2 tool. QUADAS-2, Quality Assessment of Diagnostic Accuracy Studies.

Quality assessment was performed using QUADAS-2. Based on the quality assessment graph, high-risk bias concerns were primarily discovered in flow and timing. **Figure 2** summarizes the quality of the included studies. In terms of risk of bias, four (26.7%) studies were not detailed in terms of patient selection; six (40%) studies had an unclear risk of bias for reference standards; and the flow and timing of four (26.7%) studies were judged to be vague. Two (13.3%) studies were considered to be high-risk due to extended intervals. In terms of applicability, 15 (100%) studies were judged to have low applicability. The included studies were overall considered to have a moderately low risk of bias and low concern regarding the applicability, indicating a high standard of evidence.

### 3.4 Sensitivity

**Figure 3:**
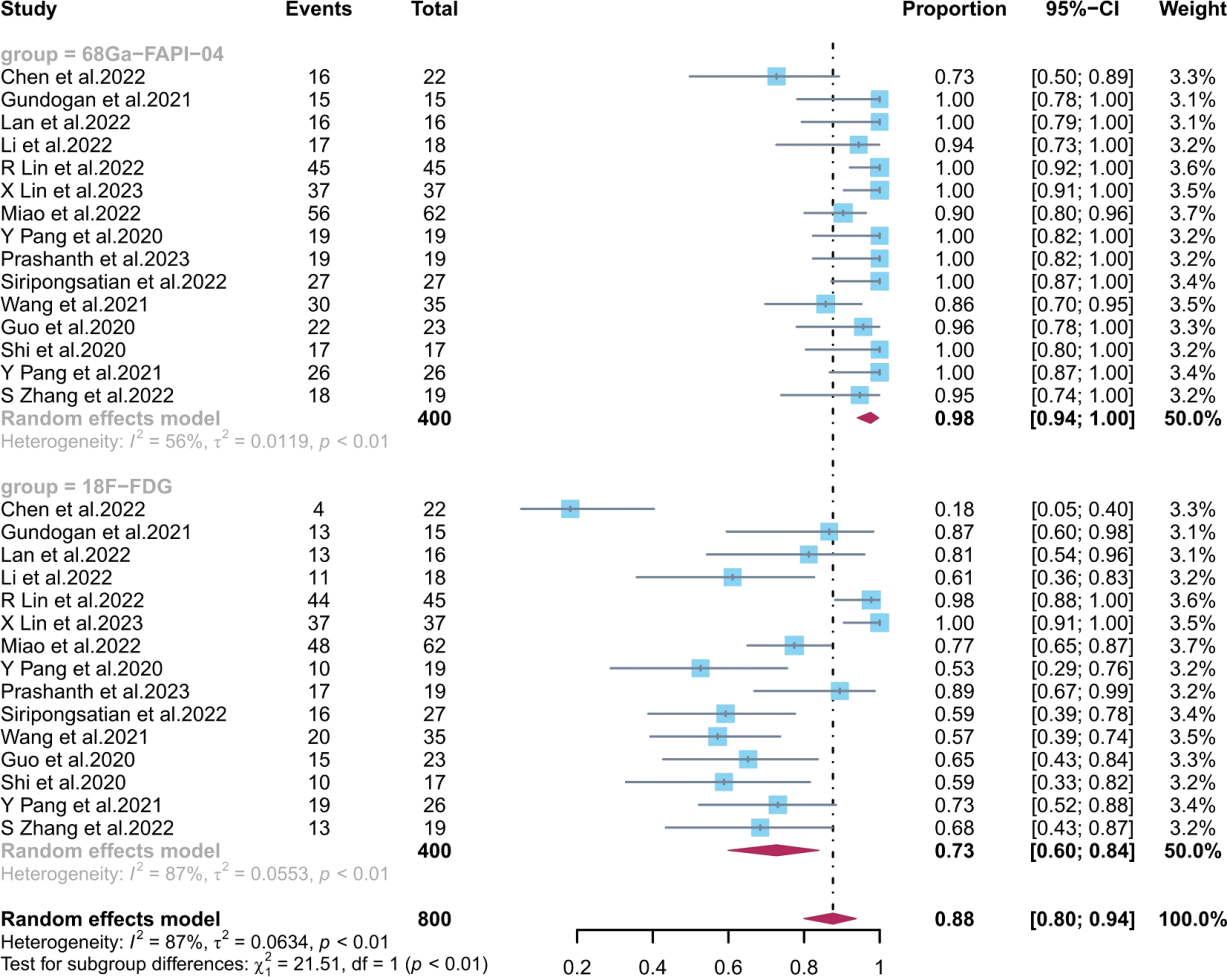
Forest plots of the combined sensitivity of ^68^Ga-FAPI-04 PET/CT and ^18^F-FDG PET/CT for digestive system cancer. FAPI, fibroblast activation protein inhibitor; PET/CT, positron emission tomography/computed tomography.

The pooled sensitivity of ^68^Ga-FAPI-04 PET/CT for primary digestive system cancer was 0.98 (95% CI, 0.94–1.00), with an I^2^ value of 56% (**Figure 3**). Meta-regression showed that the tumour stage (p = 0.009) was a possible cause of heterogeneity. Excluding the data from Chen et al., sensitivity analysis revealed a combined sensitivity of 0.98 (95% CI, 0.96–1.00), with minimal heterogeneity (I^2^ = 36.0%).

The combined ^18^F-FDG PET/CT sensitivity for primary gastrointestinal system cancer was 0.73 (95% CI, 0.60–0.84), with an I^2^ value of 87%. Meta-regression showed that the number of patients included (p = 0.04), the study design (p = 0.01), and the average size (p = 0.003) and stage (p = 0.009) of the tumours were possible causes of heterogeneity. No source of heterogeneity was identified by the sensitivity analysis for ^18^F-FDG PET/CT. The meta-regression analysis of ^68^Ga-FAPI-04 PET/CT and ^18^F-FDG PET/CT for primary digestive tract cancer is summarized in **Tables S3**, **S4** in the Supplementary Material. The sensitivity analysis of the overall detection rate for ^68^Ga-FAPI-04 PET/CT and ^18^F-FDG PET/CT is summarized in **Table S5** in the Supplementary Material.

^68^Ga-FAPI-04 PET/CT showed a significantly higher sensitivity (p < 0.01) than ^18^F-FDG PET/CT in diagnosing primary digestive system cancer.

**Figure 4:**
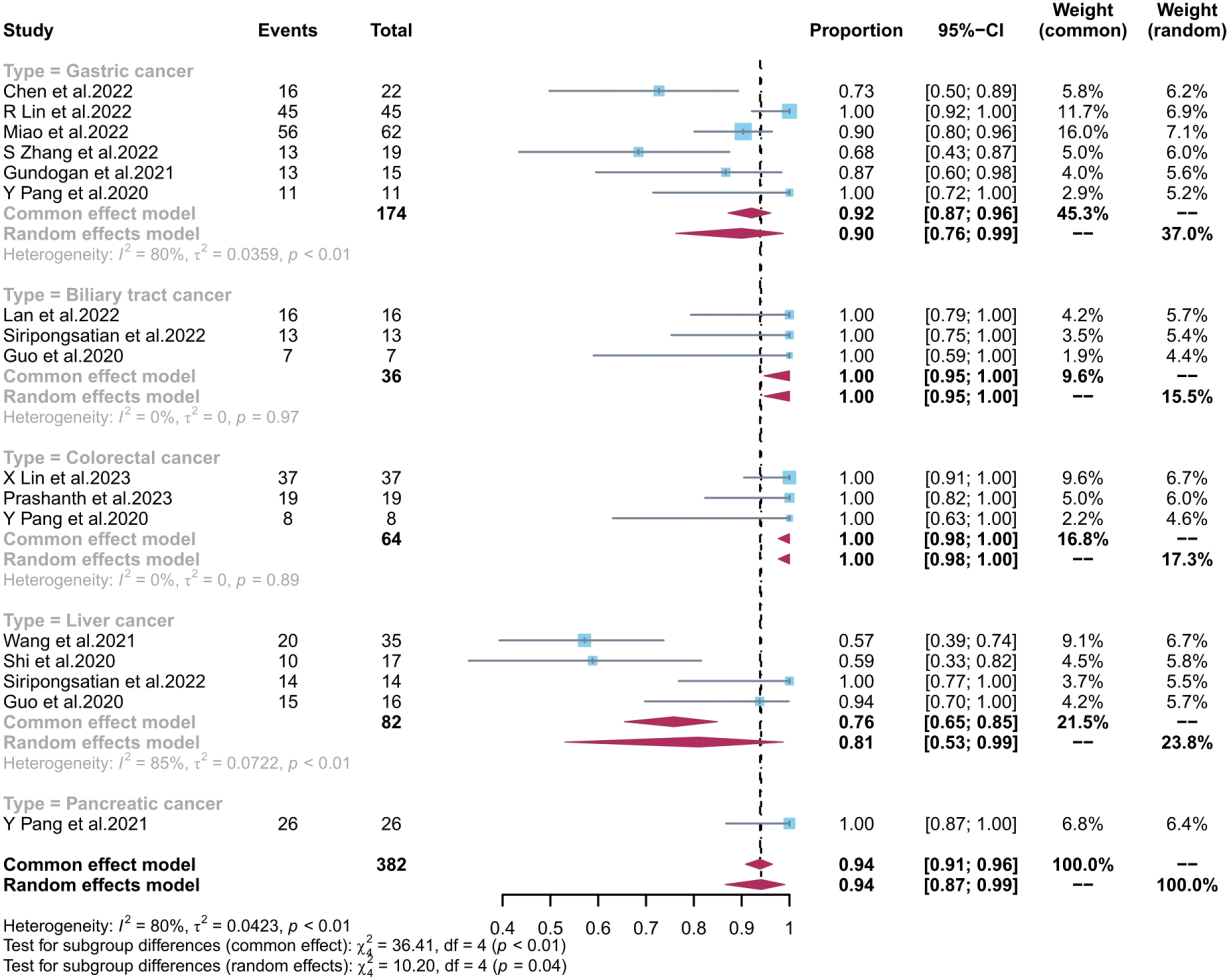
Forest plots of the combined sensitivity of ^68^Ga-FAPI-04 PET/CT with subgroups of tumour types for digestive system cancer. FAPI, fibroblast activation protein inhibitor; PET/CT, positron emission tomography/computed tomography.

**Figure 5:**
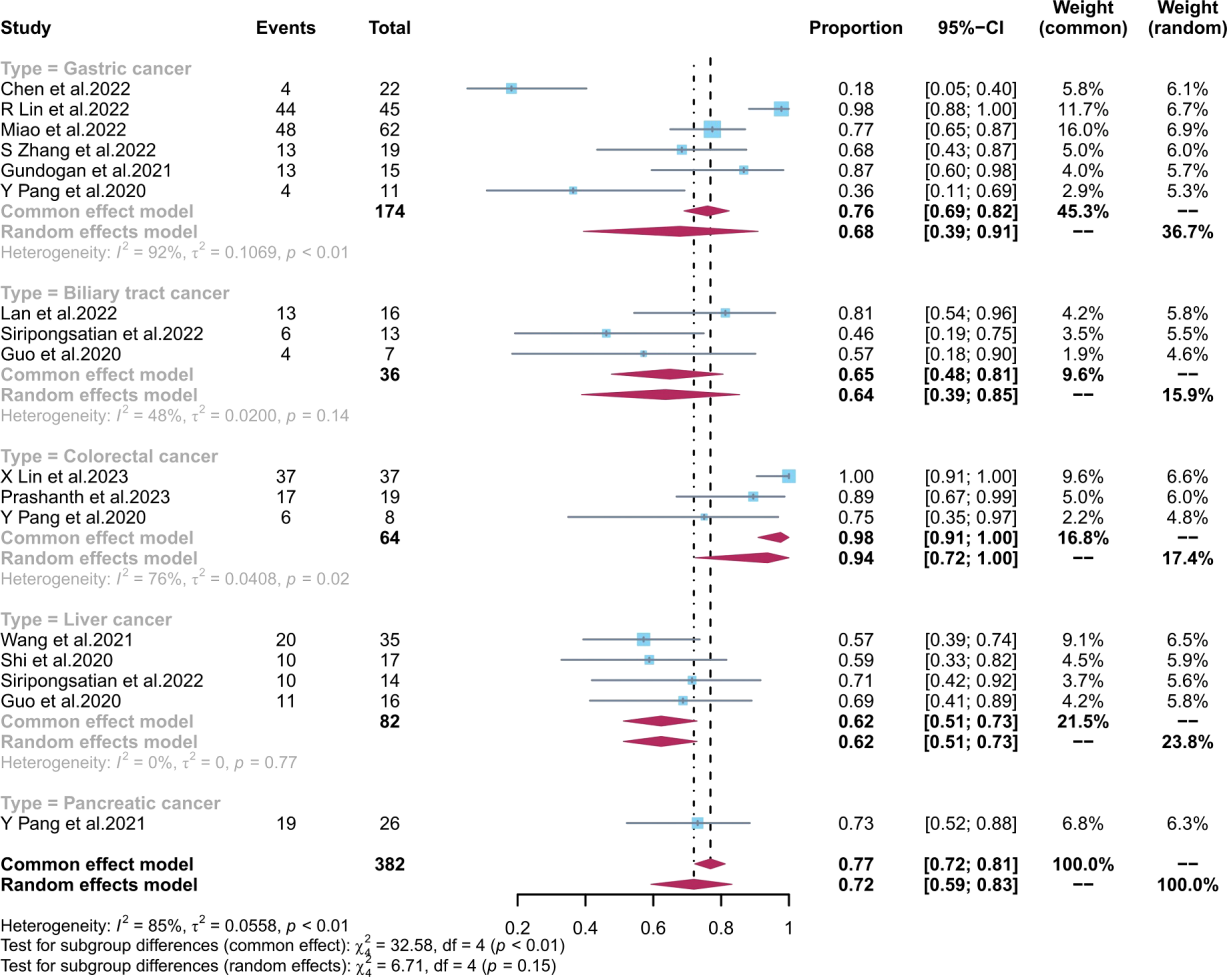
Forest plots of the combined sensitivity of ^18^F-FDG PET/CT with subgroups of tumour types for digestive system cancer. FAPI, fibroblast activation protein inhibitor; PET/CT, positron emission tomography/computed tomography.

The pooled sensitivity of ^68^Ga-FAPI-04 PET/CT and ^18^F-FDG PET/CT for GC was 0.90 (95% CI, 0.76–0.99) and 0.68 (95% CI, 0.39–0.91), respectively; for liver cancer, 0.81 (95% CI, 0.53–0.99) and 0.62 (95% CI, 0.51–0.73), respectively; for BTC, 1.00 (95% CI, 0.95–1.00) and 0.65 (95% CI, 0.48–0.81), respectively; for colorectal cancer, 1.00 (95% CI, 0.98–1.00) and 0.94 (95% CI, 0.72–1.00), respectively; and for pancreatic cancer, 1.00 (95% CI, 0.87–1.00) and 0.73 (95% CI, 0.52–0.88), respectively. There was a significant difference between the sensitivity of FAPI and FDG tracers in detecting different types of tumours (p < 0.01) (**Figures 4**, **5**). The pooled sensitivity of both ^68^Ga-FAPI-04 PET/CT and ^18^F-FDG PET/CT was the highest for colorectal cancer. The ^68^Ga-FAPI-04 PET/CT pooled sensitivity was significantly higher than that of ^18^F-FDG PET/CT for the rest of the four tumour types.

### 3.4 Specificity

The pooled specificity of ^68^Ga-FAPI-04 PET/CT for primary digestive system cancer was 0.81 (95% CI, 0.23–1.00), with an I^2^ value of 81%. Sensitivity analysis by excluding data from Pang et al. demonstrated a combined specificity of 1.00 (0.77–1.00), with no heterogeneity (I^2^ = 0%). The sensitivity analysis of overall specificity for ^68^Ga-FAPI-04 PET/CT is summarized in **Table S5** in the Supplementary Material. Due to the small number of included studies, we did not perform subgroup or meta-regression analyses.

**Figure 6:**
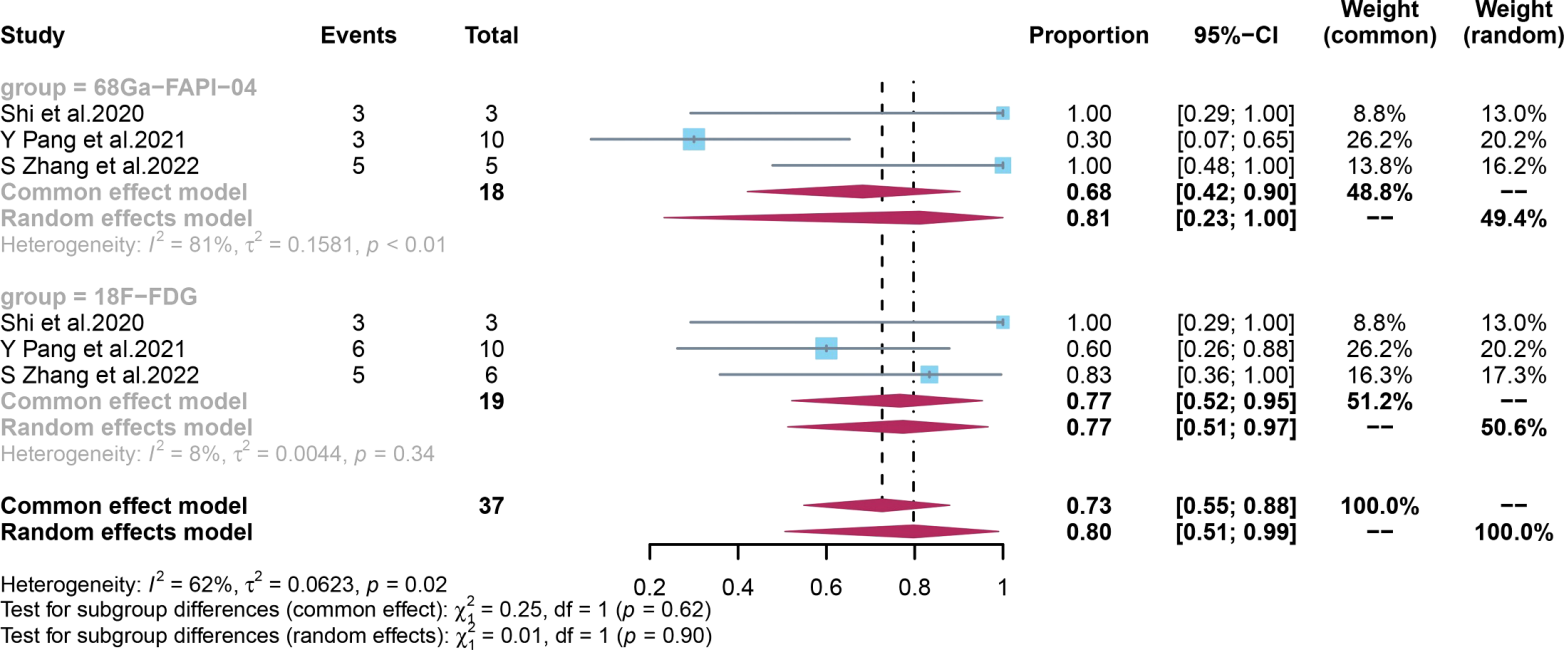
Forest plots of the combined specificity of ^68^Ga-FAPI-04 PET/CT and ^18^F-FDG PET/CT for digestive system cancer. FAPI, fibroblast activation protein inhibitor; PET/CT, positron emission tomography/computed tomography.

The pooled specificity of ^18^F-FDG PET/CT for primary digestive system cancer was 0.77 (95% CI, 0.52–0.95), with an I^2^ value of 8%, which showed low heterogeneity (**Figure 6**). No significant difference was observed in the specificities of ^68^Ga-FAPI-04 PET/CT and ^18^F-FDG PET/CT (p = 0.09).

### 3.5 Publication bias

Deek’s funnel plot asymmetry test and Egger’s test revealed no significant publication bias for ^68^Ga-FAPI-04 PET/CT (p = 0.63) and ^18^F-FDG PET/CT (p = 0.19). Deek’s funnel plot for the two contrast agents is shown in **Figures S1**, **S2** in the Supplementary Material.

## 4 Discussion

Early diagnosis of primary gastrointestinal tumours is crucial for determining a patient’s survival and recurrence risk and for developing an appropriate treatment plan. Two previous meta-analyses (42, 43) have evaluated the use of ^68^Ga-FAPI-04 PET/CT in diagnosing gastrointestinal tumours. However, neither of these studies conducted a head-to-head comparison of ^68^Ga-FAPI-04 PET/CT and ^18^F-FDG PET/CT in primary digestive tract tumours, which can provide strong evidence for choosing the most suitable contrast agent for early diagnosis. Wang et al. (42) conducted a meta-analysis comparing ^68^Ga-FAPI-04 and ^18^F-FDG PET/MRI and PET/CT studies for GC only. They reported that ^68^Ga-FAPI-04 PET/MRI or PET/CT was more effective than ^18^F-FDG PET/MRI or PET/CT in detecting primary GC. However, the statistical significance of this finding needs to be clarified. Another meta-analysis by Huang et al. (43) included fewer studies on primary digestive system cancers than our study. They reported that the sensitivity of ^68^Ga-FAPI PET for the diagnostic assessment of primary tumour lesions in the digestive system was 0.97, which is similar to our findings. However, their study did not conduct a head-to-head comparison of ^68^Ga-FAPI-04 PET/CT and ^18^F-FDG PET/CT in primary digestive tract tumours. By conducting a head-to-head comparison of ^68^Ga-FAPI-04 PET/CT and ^18^F-FDG PET/CT, our study provides strong evidence for a more suitable contrast agent for the early diagnosis of primary digestive tract tumours.

This study is the first systematic review and meta-analysis evaluating the diagnostic performance of ^68^Ga-FAPI-04 PET/CT and ^18^F-FDG PET/CT for primary digestive system cancer. The included studies were assessed as having a low risk of bias and low concern regarding applicability. The pooled sensitivity and specificity of ^68^Ga-FAPI-PET/CT vs. ^18^F-FDG PET/CT were 0.98 (95% CI, 0.94–1.00) and 0.81 (95% CI, 0.23–1.00) vs. 0.73 (95% CI, 0.60–0.84) and 0.77 (95% CI, 0.52–0.95), respectively. These results indicate that ^68^Ga-FAPI-04 PET/CT has a significantly higher sensitivity (p < 0.01) than ^18^F-FDG PET/CT in diagnosing primary digestive system cancer. However, there was no significant difference between the two contrast agents in terms of specificity. No significant publication bias was observed for either ^68^Ga-FAPI-04 PET/CT or ^18^F-FDG PET/CT. In addition, the risk of bias and concern regarding the applicability of the included studies were both low. Therefore, due to the moderately low risk of bias and low concern regarding the applicability, the certainty of the evidence was considered high.

Both the sensitivity and specificity of ^68^Ga-FAPI-PET/CT and the specificity of ^18^F-FDG PET/CT exhibited high heterogeneity. Therefore, meta-regression and sensitivity analyses were performed to identify the sources of heterogeneity among the studies. For ^68^Ga-FAPI-PET/CT, the results of the meta-regression analysis revealed that the tumour stage was a potential source of heterogeneity. Also, we achieved an acceptable level of heterogeneity (I^2^ = 36%) by eliminating data from Chen et al., whose criteria could explain the final diagnosis and cutoff values. Nonetheless, there may be additional explanations, such as patient variation, method, and analysis. Notably, the specificity of ^68^Ga-FAPI-PET/CT remained the same (1.00) when the study by Pang et al. (41) was excluded, indicating the robustness of the results. For ^18^F-FDG PET/CT, the meta-regression analysis showed that the number of patients, the study design, and the average size and stage of the tumours were possible causes of heterogeneity.

Most of the studies included in this analysis focused on GC. The pooled sensitivity of ^68^Ga-FAPI-04 PET/CT and ^18^F-FDG PET/CT for GC was 0.90 (95% CI, 0.76–0.99) and 0.68 (95% CI, 0.39–0.91), respectively. Their detection rate was [88.5% (154/174) vs. 72.4% (126/174), respectively]. Several studies (20, 21, 32) have found that FAPI PET/CT is more sensitive than FDG PET/CT in diagnosing gastric adenocarcinoma, likely due to FAPI’s ability to target fibroblasts in the tumour microenvironment with more precision. However, different pathological tumour types are associated with varying levels of FDG PET/CT uptake in GC, as noted by Jiang et al. (44) and Chen et al. (29). Miao et al. (31) and Chen et al. (29) found that FAPI was equally effective at detecting early gastric cancer (EGC) as FDG PET/CT, with both modalities having a low detection rate. Endoscopy remains the gold standard for diagnosing EGC. The sub-optimal accuracy of the FAPI tracer is reflected in its superiority to the latest National Comprehensive Cancer Network (NCCN) (45) recommendation of ^18^F-FDG PET/CT for the diagnosis of indolent cell carcinoma. For BTC, the pooled sensitivity of ^68^Ga-FAPI-04 PET/CT and ^18^F-FDG PET/CT was 1.00 (95% CI, 0.95–1.00) and 0.65 (95% CI, 0.48–0.81), respectively. The detection rate of FAPI PET/CT and FDG PET/CT for BTC was [100.00% (36/36) vs. 63.89% (23/36), respectively]. In the diagnosis and staging of intrahepatic cholangiocarcinoma and cholangiocarcinoma (CCA), FAPI is more accurate than the FDG tracer because the low liver background helps to distinguish periportal CCA from BTC invasion of the adjacent liver parenchyma. ^68^Ga-FAPI-04 diagnosis of CCA was comparable to MRI, which is becoming the gold standard in liver detection (9), with a higher target-background-ratio (TBR) for CCA as reported by Guo et al. (37). Additionally, they discovered a correlation between the severity of the primary tumor’s corresponding pathological grade and the lesion’s FAPI uptake activity. In liver cancer, the pooled sensitivity of ^68^Ga-FAPI-04 PET/CT and ^18^F-FDG PET/CT was 0.81 (95% CI, 0.53–0.99) and 0.62 (95% CI, 0.51–0.73), respectively. The detection rate of FAPI PET/CT and FDG PET/CT for liver cancer was [71.95% (59/82) vs. 62.19% (51/82)]. The FAPI tracer was able to distinguish between various types of liver nodules, unlike the FDG tracer, which is flawed in the diagnosis of primary liver cancer because well-differentiated HCC lesions have similar FDG tracer uptake capacity to healthy liver tissue. The FAPI tracer can better detect extrahepatic metastases and improve diagnostic efficiency compared with the current clinical recommendation of liver MRI (38). For colorectal cancer, we obtained results consistent with previous findings by Lin et al. (27) and Li et al. (34), showing no significant difference between the detection rate of ^68^Ga-FAPI-04 PET/CT [1.00 (95% CI, 0.98–1.00)] and ^18^F-FDG PET/CT [0.94 (95% CI, 0.72–1.00)]. However, the detection rate of FAPI and FDG for colorectal cancer was [100.00% (64/64) vs. 93.75% (60/64)]. This may be attributed to the potential of the FDG tracer to detect false negatives due to the physiological bowel activity. Based on the studies by Pang et al. (26) and Lin et al. (27), ^18^F-FDG PET/CT should be used preferentially for hypofractionated bowel cancer. Langer et al. (46) demonstrated that the clinical application of ^68^Ga-FAPI-04 PET/CT or ^18^F-FDG PET/CT diagnostics can lower the financial expenditures for patients by reducing unnecessary treatments. In previous studies, we analyzed only pancreatic cancer data from the study of Pang et al. (41) The pooled sensitivity of ^68^Ga-FAPI-04 PET/CT and ^18^F-FDG PET/CT for pancreatic cancer was 1.00 (95% CI, 0.87–1.00) and 0.73 (95% CI, 0.52–0.88), respectively. The detection rate of FAPI and FDG for pancreatic cancer was [100.00% (26/26) vs. 73.07% (19/26)]. It has been found that ^68^Ga-FAPI-04 can fill the gap in ^18^F-FDG PET/CT’s inability to detect small pancreatic tumours (<20 mm). However, ^68^Ga-FAPI-04 is less specific than ^18^F-FDG PET/CT since it has difficulty distinguishing pancreatitis from pancreatic cancer due to its affinity for inflammatory cells. Therefore, we presume that ^18^F-FDG PET/CT has a lower rate of misdiagnosis than ^68^Ga-FAPI-04 PET/CT for pancreatic cancer.

It is also important to mention the limitations of our meta-analysis. First, we searched only three databases and did not look for grey literature; in addition, we limited ourselves to English literature, thus resulting in a small sample size of included studies. Second, we could extract data on the degree of specificity of the two contrast agents only in three studies. Moreover, we excluded some studies because they only stated specificity and lacked specific TN and FP values. To clarify the difference between the two specificities, follow-up studies of high quality should be conducted to expand the sample size. Third, there was a degree of heterogeneity in the results due to the inclusion of fewer prospective than retrospective studies and the inclusion of fewer studies with large samples. The low number of high-quality studies is due to the inclusion criteria, which required head-to-head comparison studies. Thus, more prospective studies with large samples need to be conducted. Fourth, the reference standards for the diagnosis of digestive tract cancers are pathology and follow-up imaging. However, pathological results were not available for all patients in the included studies. Fifth, one of the included studies (39) involved multiple tumours, and each tumor’s TN, TP, FN, and FP values could not be extracted, leaving incomplete data for sensitivity analyses by tumour type as a subgroup. Therefore, the reported results should be interpreted with caution.

## 5 Conclusion

This study shows that ^68^Ga-FAPI-04 PET/CT has a significantly higher sensitivity (p < 0.01) than ^18^F-FDG PET/CT when used to detect primary digestive system cancer. Instead, we did not observe a significant difference in specificity between the two contrast agents. ^68^Ga-FAPI-04 PET/CT is more advantageous in diagnosing gastric, liver, biliary tract, and pancreatic cancers, while both contrast agents have the same power in diagnosing colorectal cancer. However, PET/CT results were derived from studies with small sample sizes. Therefore, our observations need to be validated by a more extensive and comprehensive prospective study.

## Data availability statement

The original contributions presented in the study are included in the article/**Supplementary Material**. Further inquiries can be directed to the corresponding author.

## Author contributions

JO: Literature Search and Review, Manuscript Writing, Meta-Analysis, Content planning. PD: Literature Search and Data collection. YL: Manuscript and Literature Search. RZ: Proofreading Manuscript. All authors contributed to the article and approved the submitted version.

## References

[1] Washington MK Goldberg RM Chang GJ Limburg P Lam AK Salto-Tellez M . Diagnosis of digestive system tumours. Int J Cancer. (2021) 148(5):1040–50. doi: 10.1002/ijc.33210 32674220

[2] Deo SVS Sharma J Kumar S . GLOBOCAN 2020 report on global cancer burden: challenges and opportunities for surgical oncologists. Ann Surg Oncol (2022) 29(11):6497–500. doi: 10.1245/s10434-022-12151-6 35838905

[3] Xia C Dong X Li H Cao M Sun D He S . Cancer statistics in China and united states, 2022: profiles, trends, and determinants. Chin Med J (Engl) (2022) 135(5):584–90. doi: 10.1097/CM9.0000000000002108 PMC8920425 35143424

[4] Fitzgerald RC Antoniou AC Fruk L Rosenfeld N . The future of early cancer detection. Nat Med (2022) 28(4):666–77. doi: 10.1038/s41591-022-01746-x 35440720

[5] Crosby D Bhatia S Brindle KM Coussens LM Dive C Emberton M . Early detection of cancer. Science (2022) 375(6586):eaay9040. doi: 10.1126/science.aay9040 35298272

[6] Sosa JA Udelsman R . Papillary thyroid cancer. Surg Oncol Clin N Am (2006) 15(3):585–601. doi: 10.1016/j.soc.2006.05.010 16882499

[7] Malherbe K Tafti D . Breast ultrasound. Treasure Island (FL: StatPearls (2023). 32491769

[8] Ellebaek SB Fristrup CW Mortensen MB . [Laparoscopic ultrasound imaging in colorectal cancer resection may increase the detection rate of small liver metastases]. Ugeskr Laeger. (2016) 178(24):1–5. 27292835

[9] Karaosmanoglu AD Onur MR Ozmen MN Akata D Karcaaltincaba M . Magnetic resonance imaging of liver metastasis. Semin Ultrasound CT MR. (2016) 37(6):533–48. doi: 10.1053/j.sult.2016.08.005 27986172

[10] Linton KD Catto JW . Whole-body magnetic resonance imaging and prostate cancer metastases: a new gold standard of detection, but does it help us and at what cost? Eur Urol (2012) 62(1):76–7. doi: 10.1016/j.eururo.2012.02.059 22424664

[11] Bisschops R East JE Hassan C Hazewinkel Y Kaminski MF Neumann H . Advanced imaging for detection and differentiation of colorectal neoplasia: European society of gastrointestinal endoscopy (ESGE) guideline - update 2019. Endoscopy. (2019) 51(12):1155–79. doi: 10.1055/a-1031-7657 31711241

[12] Mokrane FZ Lu L Vavasseur A Otal P Peron JM Luk L . Radiomics machine-learning signature for diagnosis of hepatocellular carcinoma in cirrhotic patients with indeterminate liver nodules. Eur Radiol (2020) 30(1):558–70. doi: 10.1007/s00330-019-06347-w 31444598

[13] Abe S Makiguchi ME Nonaka S Suzuki H Yoshinaga S Saito Y . Emerging texture and color enhancement imaging in early gastric cancer. Dig Endosc. (2022) 34(4):714–20. doi: 10.1111/den.14182 34716942

[14] Hafner M . Conventional colonoscopy: technique, indications, limits. Eur J Radiol (2007) 61(3):409–14. doi: 10.1016/j.ejrad.2006.07.034 17169521

[15] Rijkers AP Valkema R Duivenvoorden HJ van Eijck CH . Usefulness of f-18-fluorodeoxyglucose positron emission tomography to confirm suspected pancreatic cancer: a meta-analysis. Eur J Surg Oncol (2014) 40(7):794–804. doi: 10.1016/j.ejso.2014.03.016 24755095

[16] Ramzan A Tafti D . Nuclear medicine PET/CT gastrointestinal assessment, protocols, and interpretation. Treasure Island (FL: StatPearls (2023). 35593839

[17] Lin M Wong K Ng WL Shon IH Morgan M . Positron emission tomography and colorectal cancer. Crit Rev Oncol Hematol (2011) 77(1):30–47. doi: 10.1016/j.critrevonc.2010.04.011 20619671

[18] Koppula BR Fine GC Salem AE Covington MF Wiggins RH Hoffman JM . PET-CT in clinical adult oncology: III. gastrointestinal malignancies. Cancers (Basel) (2022) 14(11):1–34. doi: 10.3390/cancers14112668 PMC9179927 35681647

[19] Almuhaideb A Papathanasiou N Bomanji J . 18F-FDG PET/CT imaging in oncology. Ann Saudi Med (2011) 31(1):3–13. doi: 10.4103/0256-4947.75771 21245592 PMC3101722

[20] Huang S Chong H Sun X Wu Z Jia Q Zhang Y . The value of (18)F-FDG PET/CT in diagnosing pancreatic lesions: comparison with CA19-9, enhanced CT or enhanced MR. Front Med (Lausanne) (2021) 8:668697. doi: 10.3389/fmed.2021.668697 34692714 PMC8531126

[21] Annunziata S Treglia G Caldarella C Galiandro F . The role of 18F-FDG-PET and PET/CT in patients with colorectal liver metastases undergoing selective internal radiation therapy with yttrium-90: a first evidence-based review. ScientificWorldJournal. (2014) 2014:879469. doi: 10.1155/2014/879469 24672385 PMC3929576

[22] Yun M Bang SH Kim JW Park JY Kim KS Lee JD . The importance of acetyl coenzyme a synthetase for 11C-acetate uptake and cell survival in hepatocellular carcinoma. J Nucl Med (2009) 50(8):1222–8. doi: 10.2967/jnumed.109.062703 19617323

[23] Kratochwil C Flechsig P Lindner T Abderrahim L Altmann A Mier W . (68)Ga-FAPI PET/CT: tracer uptake in 28 different kinds of cancer. J Nucl Med (2019) 60(6):801–5. doi: 10.2967/jnumed.119.227967 PMC6581228 30954939

[24] Mori Y Dendl K Cardinale J Kratochwil C Giesel FL Haberkorn U . FAPI PET: fibroblast activation protein inhibitor use in oncologic and nononcologic disease. Radiology. (2023) 306(2):e220749. doi: 10.1148/radiol.220749 36594838

[25] Huang R Pu Y Huang S Yang C Yang F Pu Y . FAPI-PET/CT in cancer imaging: a potential novel molecule of the century. Front Oncol (2022) 12:854658. doi: 10.3389/fonc.2022.854658 35692767 PMC9174525

[26] Pang Y Zhao L Luo Z Hao B Wu H Lin Q . Comparison of (68)Ga-FAPI and (18)F-FDG uptake in gastric, duodenal, and colorectal cancers. Radiology. (2021) 298(2):393–402. doi: 10.1148/radiol.2020203275 33258746

[27] Lin X Li Y Wang S Zhang Y Chen X Wei M . Diagnostic value of [(68)Ga]Ga-FAPI-04 in patients with colorectal cancer in comparison with [(18)F]F-FDG PET/CT. Front Oncol (2022) 12:1087792. doi: 10.3389/fonc.2022.1087792 36698416 PMC9869033

[28] Whiting PF Rutjes AW Westwood ME Mallett S Deeks JJ Reitsma JB . QUADAS-2: a revised tool for the quality assessment of diagnostic accuracy studies. Ann Intern Med (2011) 155(8):529–36. doi: 10.7326/0003-4819-155-8-201110180-00009 22007046

[29] Chen H Pang Y Li J Kang F Xu W Meng T . Comparison of [(68)Ga]Ga-FAPI and [(18)F]FDG uptake in patients with gastric signet-ring-cell carcinoma: a multicenter retrospective study. Eur Radiol (2023) 33(2):1329–41. doi: 10.1007/s00330-022-09084-9 35976396

[30] Gundogan C Komek H Can C Yildirim OA Kaplan I Erdur E . Comparison of 18F-FDG PET/CT and 68Ga-FAPI-04 PET/CT in the staging and restaging of gastric adenocarcinoma. Nucl Med Commun (2022) 43(1):64–72. doi: 10.1097/MNM.0000000000001489 34661379

[31] Miao Y Feng R Guo R Huang X Hai W Li J . Utility of [(68)Ga]FAPI-04 and [(18)F]FDG dual-tracer PET/CT in the initial evaluation of gastric cancer. Eur Radiol (2023) 33(6):4355–66. doi: 10.1007/s00330-022-09321-1 36522509 PMC10182135

[32] Lin R Lin Z Chen Z Zheng S Zhang J Zang J . [(68)Ga]Ga-DOTA-FAPI-04 PET/CT in the evaluation of gastric cancer: comparison with [(18)F]FDG PET/CT. Eur J Nucl Med Mol Imaging. (2022) 49(8):2960–71. doi: 10.1007/s00259-022-05799-5 35462566

[33] Zhang S Wang W Xu T Ding H Li Y Liu H . Comparison of diagnostic efficacy of [(68)Ga]Ga-FAPI-04 and [(18)F]FDG PET/CT for staging and restaging of gastric cancer. Front Oncol (2022) 12:925100. doi: 10.3389/fonc.2022.925100 35847877 PMC9283765

[34] Li C Tian Y Chen J Jiang Y Xue Z Xing D . Usefulness of [(68)Ga]FAPI-04 and [(18)F]FDG PET/CT for the detection of primary tumour and metastatic lesions in gastrointestinal carcinoma: a comparative study. Eur Radiol (2023) 33(4):2779–91 doi: 10.1007/s00330-022-09251-y 36394603

[35] Wang H Zhu W Ren S Kong Y Huang Q Zhao J . (68)Ga-FAPI-04 versus (18)F-FDG PET/CT in the detection of hepatocellular carcinoma. Front Oncol (2021) 11:693640. doi: 10.3389/fonc.2021.693640 34249748 PMC8267923

[36] Shi X Xing H Yang X Li F Yao S Congwei J . Comparison of PET imaging of activated fibroblasts and (18)F-FDG for diagnosis of primary hepatic tumours: a prospective pilot study. Eur J Nucl Med Mol Imaging. (2021) 48(5):1593–603. doi: 10.1007/s00259-020-05070-9 33097975

[37] Guo W Pang Y Yao L Zhao L Fan C Ke J . Imaging fibroblast activation protein in liver cancer: a single-center *post hoc* retrospective analysis to compare [(68)Ga]Ga-FAPI-04 PET/CT versus MRI and [(18)F]-FDG PET/CT. Eur J Nucl Med Mol Imaging. (2021) 48(5):1604–17. doi: 10.1007/s00259-020-05095-0 33179149

[38] Siripongsatian D Promteangtrong C Kunawudhi A Kiatkittikul P Boonkawin N Chinnanthachai C . Comparisons of quantitative parameters of Ga-68-Labelled fibroblast activating protein inhibitor (FAPI) PET/CT and [(18)F]F-FDG PET/CT in patients with liver malignancies. Mol Imaging Biol (2022) 24(5):818–29. doi: 10.1007/s11307-022-01732-2 PMC9053129 35486293

[39] Lan L Zhang S Xu T Liu H Wang W Feng Y . Prospective comparison of (68)Ga-FAPI versus (18)F-FDG PET/CT for tumor staging in biliary tract cancers. Radiology. (2022) 304(3):648–57. doi: 10.1148/radiol.213118 35579524

[40] Prashanth A Kumar Ravichander S Eswaran P Kalyan S Maheswari Babu S . Diagnostic performance of Ga-68 FAPI 04 PET/CT in colorectal malignancies. Nucl Med Commun (2023) 44(4):276–83. doi: 10.1097/MNM.0000000000001661 36756771

[41] Pang Y Zhao L Shang Q Meng T Zhao L Feng L . Positron emission tomography and computed tomography with [(68)Ga]Ga-fibroblast activation protein inhibitors improves tumor detection and staging in patients with pancreatic cancer. Eur J Nucl Med Mol Imaging. (2022) 49(4):1322–37. doi: 10.1007/s00259-021-05576-w 34651226

[42] Wang Y Luo W Li Y . [(68)Ga]Ga-FAPI-04 PET MRI/CT in the evaluation of gastric carcinomas compared with [(18)F]-FDG PET MRI/CT: a meta-analysis. Eur J Med Res (2023) 28(1):34. doi: 10.1186/s40001-023-00997-9 36653862 PMC9847115

[43] Huang D Wu J Zhong H Li Y Han Y He Y . [(68)Ga]Ga-FAPI PET for the evaluation of digestive system tumors: systematic review and meta-analysis. Eur J Nucl Med Mol Imaging. (2023) 50(3):908–20. doi: 10.1007/s00259-022-06021-2 36326867

[44] Jiang D Chen X You Z Wang H Zhang X Li X . Comparison of [(68) Ga]Ga-FAPI-04 and [(18)F]-FDG for the detection of primary and metastatic lesions in patients with gastric cancer: a bicentric retrospective study. Eur J Nucl Med Mol Imaging. (2022) 49(2):732–42. doi: 10.1007/s00259-021-05441-w 34297193

[45] Ajani JA D'Amico TA Bentrem DJ Chao J Cooke D Corvera C . Gastric cancer, version 2.2022, NCCN clinical practice guidelines in oncology. J Natl Compr Canc Netw (2022) 20(2):167–92. doi: 10.6004/jnccn.2022.0008 35130500

[46] Langer A . A systematic review of PET and PET/CT in oncology: a way to personalize cancer treatment in a cost-effective manner? BMC Health Serv Res (2010) 10(2):1–16. doi: 10.1186/1472-6963-10-283 20932288 PMC2959014

