# Supplementary material for: Head-to-head comparison of 68Ga-FAPI-04 PET/CT and 18F-FDG PET/CT in the evaluation of primary digestive system cancer: a systematic review and meta-analysis
Source: Front Oncol. 2023 Jun 26;13:1202505. doi: 10.3389/fonc.2023.1202505 (PMC10332156; doi:10.3389/fonc.2023.1202505)
Supplement: Supplementary file 1 [file DataSheet_1.docx]

**Supplemental content for Head-to-head comparison of ^68^Ga-FAPI-04 PET/CT and ^18^F-FDG PET/CT in the evaluation of primary digestive system cancer: A systematic review and meta-analysis**

***Jiqi Ouyang, Peiwen Ding, Runshun Zhang MD, Yuexia Lu***

**Table S1.** Complete search strategy

| Database | Search |
| --- | --- |
| Pubmed | (PET OR positron emission tomography) AND (68Ga-FAPI OR FAPI-04 OR FAPI OR fibroblast activation protein OR FAP) AND (Digestive OR Gastric OR Gastrointestinal OR Pancreatic OR Pancreas OR Pancreatic OR Colorectal OR Hepatic OR Hepatocellular OR Liver) |
| Embase | ('positron emission tomography'/exp OR ‘pet’ OR 'positron emission tomography') AND (‘68Ga-FAPI’ OR ‘FAPI-04’ OR ‘FAPI’ OR ‘fibroblast activation protein’ OR ‘FAP’) AND (‘Digestive’ OR ‘Gastric’ OR ‘Gastrointestinal’ OR ‘Pancreatic’ OR ‘Pancreas’ OR ‘Pancreatic’ OR ‘Colorectal’ OR ‘Hepatic’ OR ‘Hepatocellular’ OR ‘Liver’) |
| Web of Science | (PET OR positron emission tomography) AND (68Ga-FAPI OR FAPI-04 OR FAPI OR fibroblast activation protein OR FAP) AND (Digestive OR Gastric OR Gastrointestinal OR Pancreatic OR Pancreas OR Pancreatic OR Colorectal OR Hepatic OR Hepatocellular OR Liver) |

**Table S2 Technical aspects of included studies**

| Author | Year | Mean injected activity per kg or total for FAPI | Mean injected activity per kg or total for FDG | Time interval FAPI injection and image acquisition | Time interval FDG injection and image acquisition | Median period between FAPI and FDG（range） | Scanner Modality |
| --- | --- | --- | --- | --- | --- | --- | --- |
| H Chen et al. | 2022 | 194.3MBq (range133.2–281.2) | 281.2 MBq (range, 203.5–358.9) | 60min | 60min | 2 days ( 1–7 days) | Biograph mCT- Siemens Healthineers |
| Gundogan et al. | 2021 | 2 MBq/kg | 3.5–5.5 MBq/kg | 60min | 60min | 7 days | GE  Healthcare, Milwaukee, Wisconsin, USA |
| L Lan et al. | 2022 | 1.85 MBq/kg | 3.7 MBq/kg | 45-60min | 45-60min | 3 days | uMI780, United Imaging Healthcare |
| C Li et al. | 2022 | 1.85-3.70 MBq/kg | 3.70-5.55 MBq/kg | 40-60min | 40-60min | 1 days | Biograph mCT- Siemens Healthineers |
| R Lin et al. | 2022 | 111–185 MBq | 3.7 MBq/kg | 35–71min | 35–71min | ＜7 days | Biograph mCT64, Siemens Healthcare |
| X Lin et al. | 2023 | 1.85–2.96MBq/kg | NA | 60min | 60min | NA | Biograph mCT Flow 64; Siemens  Healthineers USA, Knoxville, TN, USA |
| Miao et al. | 2022 | 1.85–2.96 MBq | 3.7–4.44 Mbq | 30–60 min | 30–60 min | ＜9 days | Biograph Vision 450,  Siemens Healthineers |
| Y Pang et al. | 2020 | 1.8–2.2 MBq(0.1mCi)/kg | 3.7 MBq(0.05–0.06 mCi)/kg | 60min | 60min | 2 days ( 1–6 days) | Discovery MI; GE Health-  care |
| Prashanth et al. | 2023 | 1.8–2.2 MBq (0.05–0.06mCi)/kg | 3.7 MBq (0.1 mCi)/kg | 20min | 60min | 1 days (1–3 days) | Discovery MI; GE Health-  care |
| Siripongsatian et al. | 2022 | 2.59 MBq/kg | 2.59 MBq/kg | 60min | 60min | ＜7 days | Siemens Healthineers,  Erlangen, Germany |
| H Wang et al. | 2021 | 185MBq | NA | 60min | 60min | 1 day | mMI510, Union imaging, Shanghai, China |
| Guo et al. | 2020 | 259MBq | 3.7 MBq (0.1 mCi/kg) | 60min | 60min | 7 days | Discovery MI, GE Healthcare, Milwaukee, WI, USA |
| Shi et al. | 2020 | 196-260 MBq (3.59 ± 0.47 MBq/kg) | 3.7 MBq/kg, | 90min | 90min | 3 days | PoleStar m660, Sinounion Healthcare  Inc., Beijing, China |
| Y Pang et al. | 2021 | 3.7 MBq (0.1mCi)/kg | 1.8–2.2 MBq (0.05–0.06 mCi)/kg | 60min | 60min | 7 days | Discovery MI, GE Healthcare,  Milwaukee, WI, USA |
| S Zhang et al. | 2022 | 3.7 MBq (0.1 mCi/kg) | 3.7 MBq/kg | 60min | 60min | 7 days | uMI780, United Imaging Healthcare |

**Table S3.** Subgroup analysis and meta-regression of ^68^GaFAPI-04 PET/CT in the evaluation of primary digestive tract cancer

| Covariate/Subgroup | Studies, n | Sensitivity(95%CI) | P-value |
| --- | --- | --- | --- |
| Number of patients included |  |  | 0.18 |
| ＞25 | 5 | 0.99(0.95-1.00) |  |
| ≤25 | 10 | 0.96(0.91-1.00) |  |
| Ethnicity |  |  | 0.64 |
| Asian | 14 | 0.97(0.94-1.00) |  |
| the rest | 1 | 1.00(0.78-1.00) |  |
| Study design |  |  | 0.10 |
| Retrospective | 8 | 0.95(0.88-0.99) |  |
| Prospective | 7 | 0.99(0.96-1.00) |  |
| Criteria for final diagnosis |  |  | 1.00 |
| PA | 4 | 0.98(0.89-1.00) |  |
| PA or FU | 11 | 0.98(0.93-1.00) |  |
| Analysis |  |  | 0.73 |
| Patients | 10 | 0.98(0.94-1.00) |  |
| Lesions | 5 | 0.97(0.89-1.00) |  |
| Average Size（cm） |  |  | 0.52 |
| ＜3 | 2 | 0.91(0.51-1.00) |  |
| ≥3 | 3 | 0.95(0.90-0.99) |  |
| Average Age（years） |  |  | 0.23 |
| ＜60 | 1 | 0.94(0.89-0.98) |  |
| ≥60 | 3 | 0.98(0.89-1.00) |  |
| Gender (M%) |  |  | 0.25 |
| ＜70% | 3 | 0.99(0.94-1.00) |  |
| ≥70% | 3 | 0.93(0.82-1.00) |  |
| Stage(Early:Advanced) |  |  | 0.009 |
| ＜1:1 | 5 | 0.98(0.93-1.00) |  |
| ≥1:1 | 2 | 0.81(0.70-0.91) |  |

**Table S4.** Subgroup analysis and meta-regression of ^18^F-FDG PET/CT in the evaluation of primary digestive tract cancer

| Covariate/Subgroup | Studies, n | Sensitivity(95%CI) | P-value |
| --- | --- | --- | --- |
| Number of patients included |  |  | 0.04 |
| ＞25 | 5 | 0.86(0.66-0.98) |  |
| ≤25 | 10 | 0.68(0.50-0.77) |  |
| Ethnicity |  |  | 0.56 |
| Asian | 14 | 0.72(0.58-0.84) |  |
| the rest | 1 | 0.73(0.60-0.98) |  |
| Study design |  |  | 0.01 |
| Retrospective | 8 | 0.59(0.44-0.73) |  |
| Prospective | 7 | 0.86(0.72-0.96) |  |
| Criteria for final diagnosis |  |  | 0.97 |
| PA | 4 | 0.72(0.52-0.89) |  |
| PA or FU | 11 | 0.73(0.56-0.87) |  |
| Analysis |  |  | 0.16 |
| Patients | 10 | 0.66(0.50-0.80) |  |
| Lesions | 5 | 0.84(0.65-0.97) |  |
| Average Size（cm） |  |  | 0.003 |
| ＜3 | 2 | 0.58(0.36-0.79) |  |
| ≥3 | 3 | 0.74(0.65-0.82) |  |
| Average Age（years） |  |  | 0.24 |
| ＜60 | 1 | 0.70(0.56-0.82) |  |
| ≥60 | 3 | 0.74(0.60-0.87) |  |
| Gender (M%) |  |  | 0.37 |
| ＜70% | 3 | 0.75(0.63-0.86) |  |
| ≥70% | 3 | 0.66(0.51-0.79) |  |
| Stage(Early:Advanced) |  |  | 0.009 |
| ＜1:1 | 5 | 0.82(0.62-0.93) |  |
| ≥1:1 | 2 | 0.37(0.06-0.76) |  |

| **Table S5.** Sensitivity analysis of overall detection rate for ^68^Ga-FAPI-04PET/CT and ^18^F-FDG PET/CT | | | | | |
| --- | --- | --- | --- | --- | --- |
|  | ^68^Ga-FAPI-04 PET/CT | |  | ^18^F-FDG PET/CT | |
|  | Sensitivity  (95%CI) | I2 |  | Sensitivity  (95%CI) | I2 |
| Omitting Chen et al. 2022 | 0.98(0.96-1.00) | 36.50% |  | 0.76(0.60-0.84) | 81.80% |
| Omitting Gundogan et al. 2021 | 0.97(0.94-1.00) | 58.90% |  | 0.72(0.66-0.86) | 87.40% |
| Omitting Lan et al. 2022 | 0.97(0.94-1.00) | 58.80% |  | 0.72(0.58-0.84) | 87.50% |
| Omitting Li et al. 2022 | 0.98(0.94-1.00) | 59.00% |  | 0.73(0.58-0.84) | 87.30% |
| Omitting R Lin et al.2022 | 0.97(0.93-1.00) | 54.10% |  | 0.70(0.60-0.85) | 83.70% |
| Omitting X Linet all. 2023 | 0.97(0.93-1.00) | 55.70% |  | 0.69(0.57-0.81) | 81.80% |
| Omitting Miao et al. 2022 | 0.98(0.95-1.00) | 53.80% |  | 0.72(0.57-0.80) | 87.50% |
| Omitting Y Pang etal. 2020 | 0.97(0.94-1.00) | 56.20% |  | 0.74(0.58-0.84) | 87.00% |
| Omitting Prashanth et al. 2023 | 0.97(0.94-1.00) | 58.50% |  | 0.71(0.61-0.85) | 87.30% |
| Omitting Siripongsatian et al. 2022 | 0.97(0.93-1.00) | 57.50% |  | 0.74(0.58-0.83) | 87.10% |
| Omitting Wang et al. 2021 | 0.98(0.95-1.00) | 50.90% |  | 0.74(0.60-0.85) | 86.80% |
| Omitting Guo et al. 2020 | 0.98(0.94-1.00) | 59.30% |  | 0.73(0.60-0.86) | 87.40% |
| Omitting Shi et al. 2020 | 0.97(0.94-1.00) | 58.70% |  | 0.74(0.60-0.85) | 87.30% |
| Omitting Y Pang et al. 2021 | 0.97(0.93-1.00) | 58.50% |  | 0.73(0.60-0.85) | 87.50% |
| Omitting S Zhang et al. 2022 | 0.98(0.94-1.00) | 59.10% |  | 0.73(0.59-0.85) | 87.50% |

**Table S6.** Sensitivity analysis of overall specificity for  ^68^Ga-FAPI-04 PET/CT

|  | Sensitivity(95%CI) | I2 |
| --- | --- | --- |
| Omitting Shi et al.2020 | 0.70(0.00-1.00) | 88.10% |
| Omitting Y Pang et al.2021 | 1.00(0.77-1.00) | 0.00% |
| Omitting S Zhang et al.2022 | 0.66(0.00-1.00) | 81.10% |

**
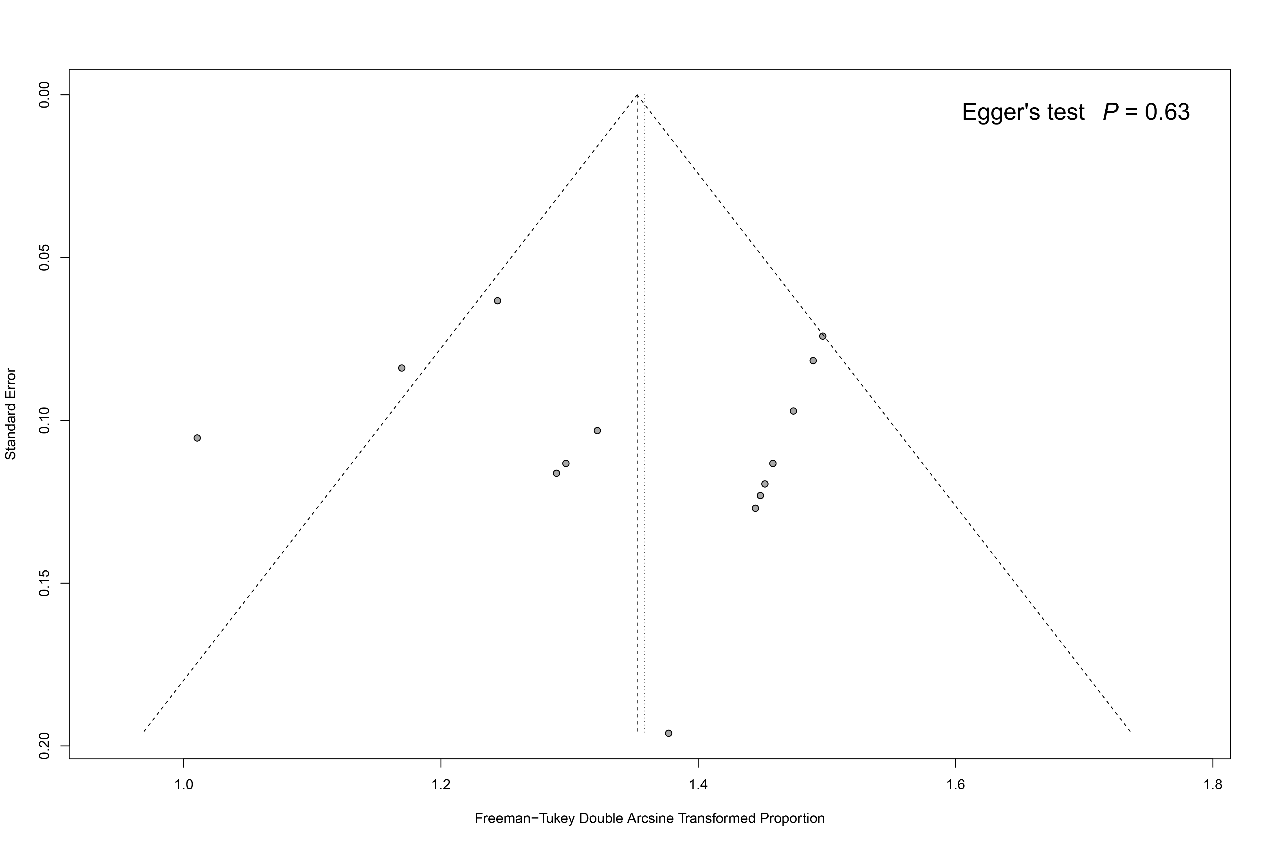
**

**Fig S1.** The Deeks' funnel plot of ^68^Ga-FAPI-04 PET/CT.( P＜0.05 was considered significant.)

**
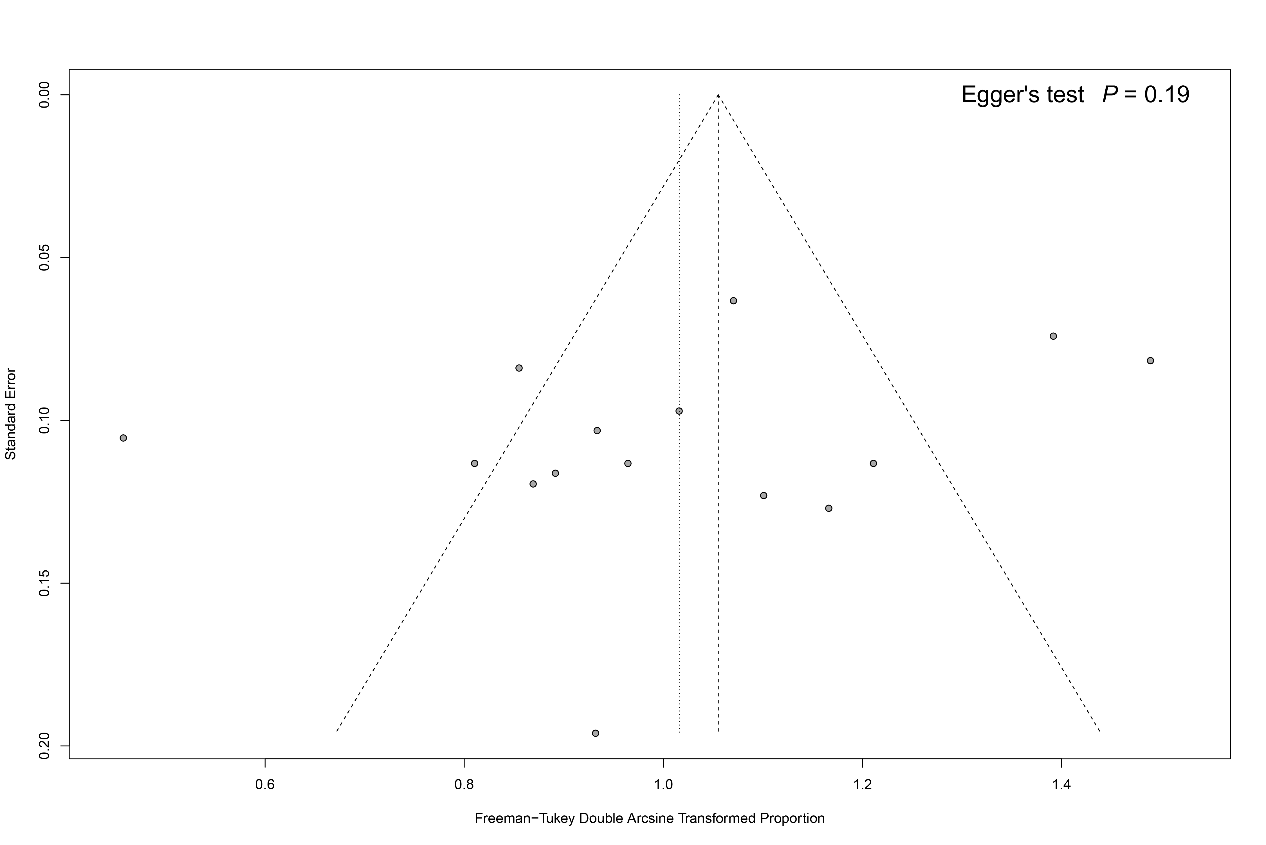
**

**Fig S2.** The Deeks' funnel plot of ^18^F-FDG PET/CT.( P＜0.05 was considered significant.)
